# Inoculum Concentration Influences Pseudomonas aeruginosa Phenotype and Biofilm Architecture

**DOI:** 10.1128/spectrum.03131-22

**Published:** 2022-11-10

**Authors:** Mads Lichtenberg, Lasse Kvich, Sara Louise Borregaard Larsen, Tim Holm Jakobsen, Thomas Bjarnsholt

**Affiliations:** a Costerton Biofilm Center, Department of Immunology and Microbiology, Faculty of Health and Medical Sciences, University of Copenhagen, Denmark; b Department of Clinical Microbiology, Copenhagen University Hospital-Rigshospitalet, Copenhagen, Denmark; University of Manitoba

**Keywords:** alginate bead, biofilm, microenvironment, oxygen, *Pseudomonas aeruginosa*, model system, spatial structure

## Abstract

In infections, bacterial cells are often found as relatively small multicellular aggregates characterized by a heterogeneous distribution of phenotype, genotype, and growth rates depending on their surrounding microenvironment. Many laboratory models fail to mimic these characteristics, and experiments are often initiated from planktonic bacteria given optimal conditions for rapid growth without concerns about the microenvironmental characteristics during biofilm maturation. Therefore, we investigated how the initial bacterial concentration (henceforth termed the inoculum) influences the microenvironment during initial growth and how this affects the sizes and distribution of developed aggregates in an embedded biofilm model—the alginate bead biofilm model. Following 24 h of incubation, the viable biomass was independent of starting inoculum but with a radically different microenvironment which led to differences in metabolic activity depending on the inoculum. The inoculum also affected the number of cells surviving treatment with the antibiotic tobramycin, where the highest inoculum showed higher survival rates than the lowest inoculum. The change in antibiotic tolerance was correlated with cell-specific RNA content and O_2_ consumption rates, suggesting a direct role of metabolic activity. Thus, the starting number of bacteria results in different phenotypic trajectories governed by different microenvironmental characteristics, and we demonstrate some of the possible implications of such physiological gradients on the outcome of *in vitro* experiments.

**IMPORTANCE** Biofilm aggregates grown in the alginate bead biofilm model bear resemblance to features of *in vivo* biofilms. Here, we show that changing the initial concentration of bacteria in the biofilm model leads to widely different behavior of the bacteria following an incubation period. This difference is influenced by the local conditions experienced by the bacteria during growth, which impact their response to antibiotic treatment. Our study provides a framework for manipulating aggregate sizes in *in vitro* biofilm models. It underlines the importance of how experiments are initiated, which can profoundly impact the outcomes and interpretation of microbiological experiments.

## INTRODUCTION

It is well established that the biofilm mode of growth, i.e., aggregated bacteria encapsulated in an extracellular matrix, is the preferred mode of bacterial growth, regardless of habitat (1). In most soft tissue infections, biofilm aggregates range from 5 to 200 μm (2). What governs this size distribution is still not fully understood (3), but studies have shown that fast-growing cell clusters experience a diffusion limitation of respiratory electron acceptors and nutrients (4), potentially limiting growth. In addition to the oxygen limitation caused by bacterial metabolism, the establishment of a hypoxic or anoxic environment is further exacerbated by the high presence of polymorphonuclear neutrophils (PMNs) that consumes oxygen for use in their oxidative burst, further limiting the growth of opportunistic bacterial pathogens such as Pseudomonas aeruginosa (3, 5). Previous studies have shown that the bacterial contribution to O_2_ limitation is diminutive in endobronchial secretions from long-term infected cystic fibrosis patients (6), and an inverse correlation between the growth rate of P. aeruginosa and the concentration of PMNs was previously shown *in vivo*, highlighting their restrictive growth properties (7). However, in the absence of other oxygen consumers, bacteria can limit their own oxygen availability by exhibiting high metabolic activity (8).

Although *in vitro* investigations are highly simplified compared with the growth of bacteria in the *in vivo* infectious microenvironment (9), physicochemical factors govern the growth in both cases. Multiple variables shape the microenvironment of and around biofilms (3), and changes in the microenvironment will impact the growth rate (4). Smaller aggregates are less diffusion-limited than larger aggregates, although phenotypic and chemical gradients can occur within micrometer distances in small aggregates (10). Changes in the population size can lead to hypoxic or anoxic microenvironments, which in turn change the oxygen gradient by changing the cell-specific oxygen uptake rate (11). Large biofilms display a heterogeneous microenvironment, given a steady substrate supply, where bacteria experience physiological gradients and a diverse range of growth states (12), indicating a reduced fitness for growth-limited subpopulations. However, the distinct growth states have the potential to be beneficial for the community. For example, treatment with antibiotics only targeting actively growing bacteria result in the survival of the nonactive part of the bacterial population (5, 13). Whether subpopulations exist in *in vivo* biofilm aggregates is not clear (14). Most types of antibiotics (e.g., aminoglycosides, quinolones, and beta-lactams) target metabolically active processes, such as protein and peptide synthesis and DNA replication, and are, as such, dependent on active growth (15), which, to a large extent, is controlled by the availability of respiratory electron acceptors. In addition, the function of some types of antibiotics is directly linked to O_2_ availability (16, 17). Thus, the microenvironment and the metabolic state of cells in a biofilm influence the efficacy of antibiotic therapy (15, 18–20).

In many microbiological laboratory experiments, the outcomes of, e.g., antibiotic treatment, are assessed following 24 h of incubation without concerns about the metabolic trajectory bacteria experience before treatment. Models are often started from planktonic bacteria to ensure optimal growth conditions and reproducibility, and little notice is given to the physicochemical environment during this first “biofilm maturation” period. It has previously been shown that the inoculation method can influence the amount and size of aggregates in liquid batch cultures, which affects antibiotic tolerance (21). Even in high-throughput methods such as the microtiter assay, minor variations can lead to stochastic results (22).

**FIG 1:**
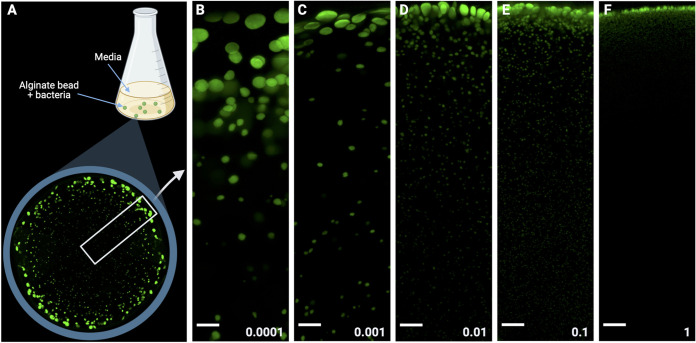
(A) Conceptual representation of the alginate beads containing bacterial aggregates. Alginate beads containing bacteria are produced by extrusion, dropping an alginate/bacterial solution into a stirred CaCl_2_ solution. The droplets solidify into spherical beads upon contact with the CaCl_2_ solution. After solidification, alginate beads were incubated in shaken Erlenmeyer flasks containing R2A media. The zoom-in shows a composite image of an alginate bead cross-section with the formed bacterial aggregates, and the rectangle depicts a transect used for image analysis. (B to F) Representative confocal microscope images of the size and distribution of bacterial aggregates in the alginate beads (scalebars = 100 μm). The models were inoculated with increasing amounts of bacteria by optical density (OD) adjustment to a final OD_450nm_ of 0.0001, 0.001, 0.01, 0.1, and 1. The images shown are z-projections of image stacks and were gamma adjusted to visualize aggregates at the bottom of the stack.

Here, we have investigated how the size and distribution of aggregates are influenced by the starting concentration of bacteria (the inoculum). We do this in the alginate bead biofilm model which aims at simulating the *in vivo* spatial distribution of aggregates in soft tissue infections where bacteria are embedded in a secondary matrix (23) (Fig. 1). We hypothesize that the density and distribution of bacteria will change the electron acceptor availability during the development of individual aggregates. As an example, we highlight the importance of oxygen availability in relation to biomass distribution. We show that the microenvironment is highly dynamic during initial growth and governs the outcomes in the biofilm models under investigation.

## RESULTS

### The inoculum alters biofilm architecture but not the total biomass.

**FIG 2:**
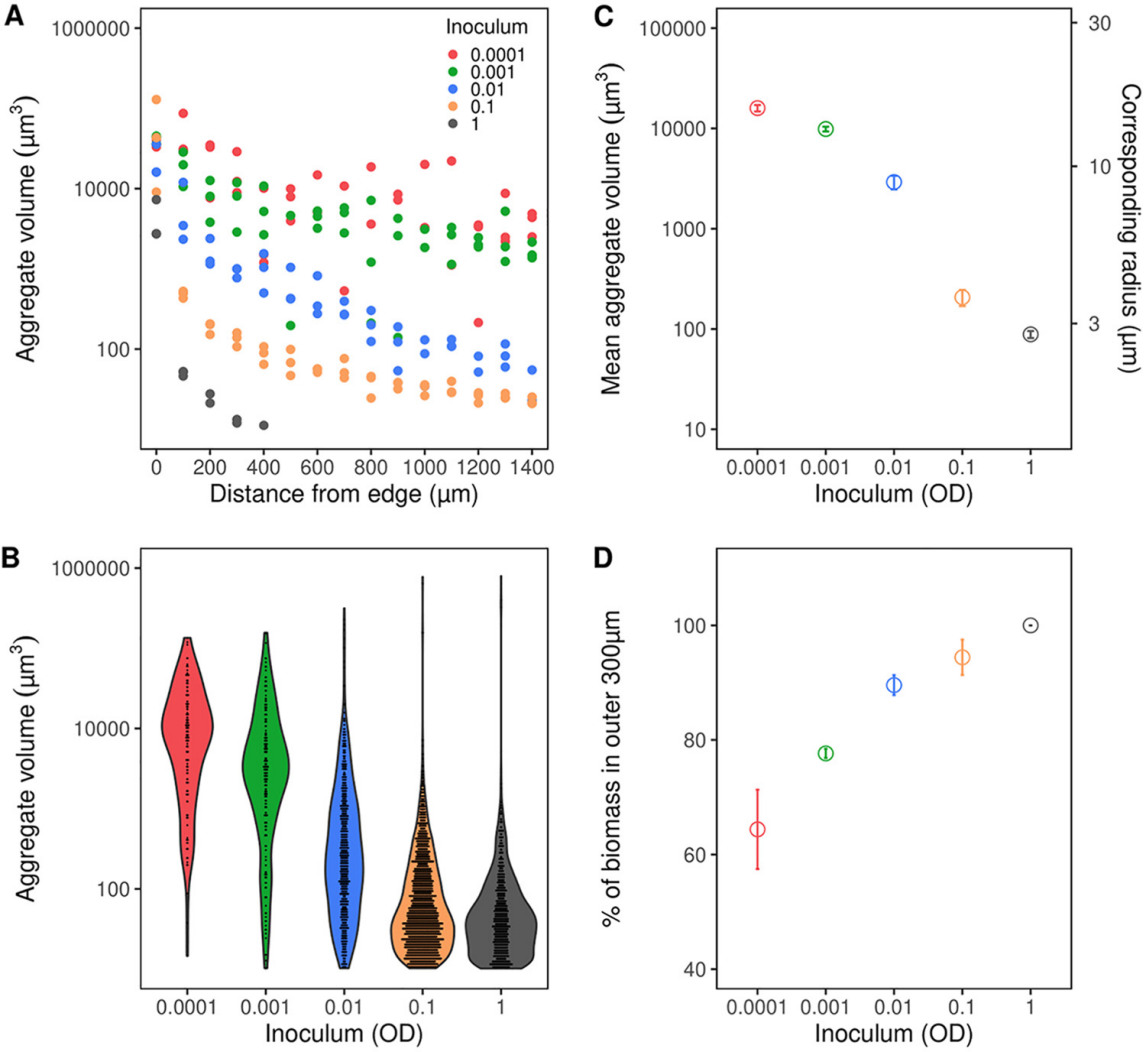
(A) Aggregate volumes as a function of distance from the bead edge between the different inoculums. Data were binned into 100-μm intervals, and the average aggregate volume for bins is shown for each replicate. (B) Density plots showing aggregate volume distribution as a function of inoculum. (C) Mean aggregate volumes and the corresponding radius (assuming spherical aggregates) in alginate beads inoculated with an increasing number of bacteria. (D) The proportion of the biomass (in %) associated with the outer 300 μm of the model as a function of inoculum. Data are presented as means ± SEM; *n* = 3.

Different concentrations of single cells were immobilized in the semisolid-hydrogel alginate matrix at t0, which resulted in bacterial aggregates of different sizes developed in the alginate beads after 24 h of incubation (Fig. 1). The mean size of aggregates in the alginate beads was dependent on inoculum, where higher inoculums led to smaller mean size of aggregates, while lower inoculums led to larger mean size of aggregates. Within each inoculum, aggregate volumes were not uniformly distributed throughout the models but were larger near the edge, followed by a rapid decrease in volume toward the center of the models. The rapid decrease in volume toward the center became less pronounced in the lower inoculums (Fig. 2A), where larger aggregates were observed in the center. The frequency distribution of aggregate volumes changed with inoculum, where low inoculums led to a higher proportion of large aggregates, while high inoculums led to a higher proportion of small aggregates (Fig. 2B).

In the lower inoculums, the aggregates were larger and more distanced than in the higher inoculums, where aggregates were more densely packed, and the volume of most aggregates was less than 1,000 μm^3^, corresponding to a radius of 6.2 μm, assuming sphericity (Fig. 2C).

Furthermore, the location of aggregates was not distributed uniformly but was associated with the edge of the models, where an increase in inoculum led to a successively larger proportion of the biomass located near the edge (Fig. 2D).

**FIG 3:**
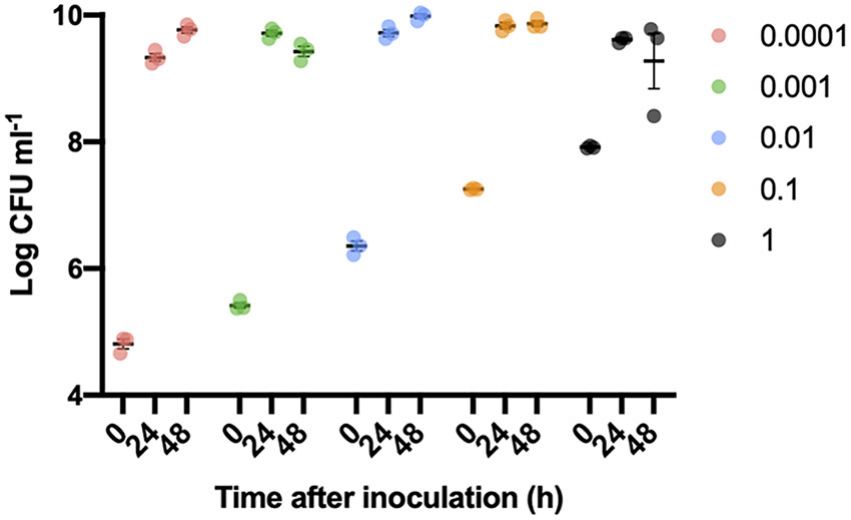
Development of viable bacteria in alginate beads inoculated with increasing amounts of bacteria. Samples were taken 0 h, 24 h, and 48 h after inoculation and were enumerated by counting CFU per milliliter. The legend indicates the inoculum measured by optical density (OD_450nm_). CFU/mL was log-transformed, and results are presented as means ± SEM; *n* = 3.

The10- to 10,000-fold increase in bacterial inoculum in the models was significantly different from each other (*P* < 0.05). However, the final number of viable bacteria was not dependent on the inoculum following 24 h and 48 h of incubation (*P* > 0.05). Thus, the total biomass in the models was not different between inoculums after 24 h (Fig. 3).

### The inoculum influences bacterial metabolic activity and can result in heterogeneous subpopulations.

**FIG 4:**
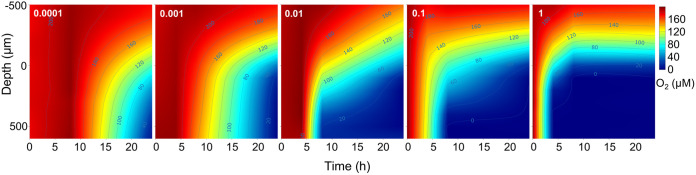
Isopleths of O_2_ concentration as a function of depth in the alginate beads and time of inoculation. Zero on the *y* axis indicates the alginate bead surface. Negative values are measurements outside the bead, while positive y values are measurements inside the bead. The color bar shows O_2_ concentration (in μM). Measurements were taken at 0 h, 4 h, 8 h, and 24 h. Numbers in the top left corners indicate the inoculum measured by optical density (OD_450nm_). Results are presented as means; *n* = 3.

**FIG 5:**
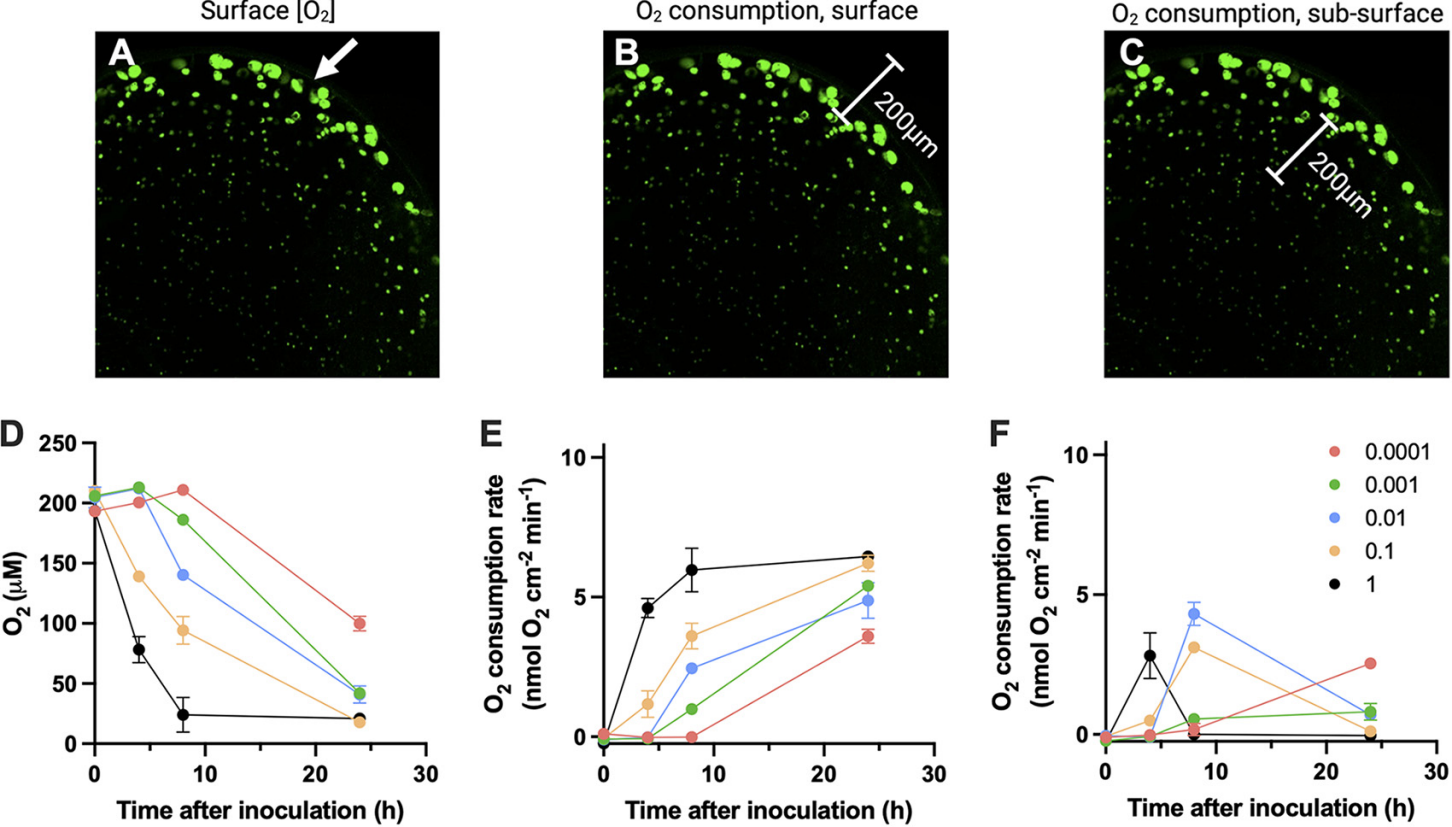
Graphical depiction of the position of measurements of (A) O_2_ concentration at the surface of the alginate beads; (B) O_2_ consumption rates at the surface (C) and subsurface O_2_ consumption rates; (D) O_2_ concentration at the surface of the alginate beads as a function of time and inoculum; (E) O_2_ consumption rates of all inoculums at the alginate beads’ surface as a function of time; (F) subsurface O_2_ consumption rates of the alginate beads as a function of time. The O_2_ consumption rates were calculated from the measured O_2_ concentration profiles. Numbers in the legend indicate the inoculum measured by optical density (OD_450nm_). Results are presented as means ± SEM; *n* = 3.

We assessed the metabolic activity of P. aeruginosa embedded in the alginate beads in three separate experiments. First, microscale oxygen concentration profiles were both time- and inoculum-dependent (Fig. 4). A change in O_2_ concentration was detected after 4 h for the two highest inoculums where the interior of the beads of the highest inoculum was already anoxic. After 8 h of incubation, all alginate beads showed decreased internal O_2_ concentrations that were correlated with the inoculum, except for the lowest inoculum (OD_450nm_ = 0.0001), where O_2_ was still fully present inside (Fig. 4). After 24 h of incubation, all inoculums showed anoxic conditions in the interior of the beads (Fig. 4). The O_2_ concentration at the surface of the models decreased over time and was dependent on the initial inoculum, where the highest inoculums showed a rapid decrease in surface O_2_ concentration (Fig. 5A and D). The surface O_2_ concentration after 24 h was dependent on inoculum, where the highest inoculums (1 and 0.1) led to microaerobic conditions while the surface of lower inoculums was still oxic (>100 μM O_2_ in the lowest inoculum; Fig. 5D). Surface O_2_ consumption rates (in nmol O_2_ cm^−2 ^min^−1^) showed an inverse relationship to the surface O_2_ concentration and increased over time in all inoculums (Fig. 5B and E). All inoculums sustained high O_2_ consumption rates near the surface of the models after 24 h. However, the subsurface O_2_ consumption rates varied between inoculums, where the highest inoculum of the alginate beads peaked after 4 h and decreased toward zero after 8 h (Fig. 5C and F). The inoculums, 0.1 and 0.01, peaked after 8 h with a following decrease, whereas the two lowest inoculums (0.001 and 0.0001) increased continuously (Fig. 5F).

**FIG 6:**
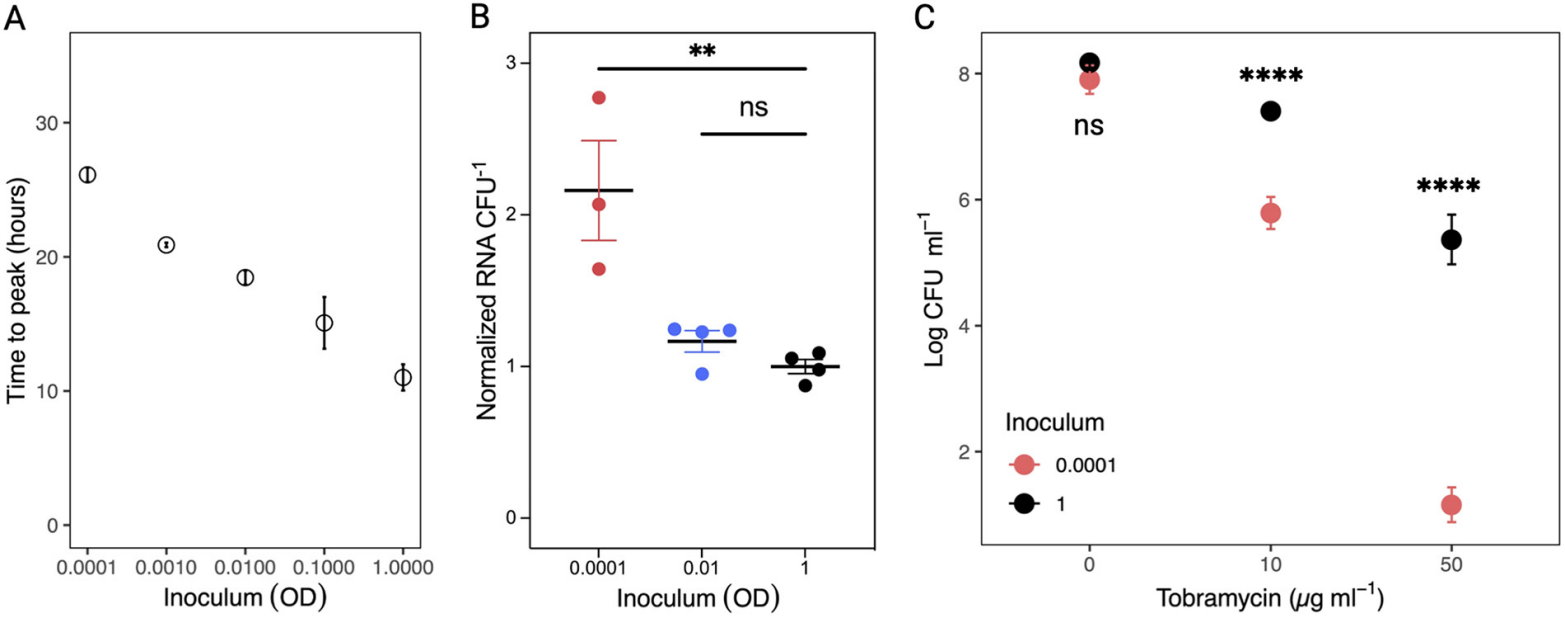
(A) Time to peak metabolic activity measured by microcalorimetry for alginate beads inoculated with an increasing number of bacteria (OD adjusted). Time to peak increased linearly with (log-transformed) inoculum (*P* < 0.05; linear regression analysis). (B) Total RNA content in alginate beads per CFU for inoculums 0.0001, 0.01, and 1. Data are normalized to OD = 1. Results are presented as means ± SEM; *n* = 4 (OD = 1 and OD = 0.01), *n* = 3 (OD = 0.0001). (C) Effect of inoculum on bacterial survival after treatment with tobramycin. Graph shows surviving bacteria following 24 h of exposure to 0, 10, or 50 μg mL^−1^ tobramycin. The controls (0 μg mL^−1^) were not statistically different, meaning the same number of bacteria were treated. Log reduction of viable bacteria was higher for the low inoculum upon exposure to 10 and 50 μg mL^−1^ tobramycin (*P* < 0.0001). Results are presented as means ± SEM; *n* = 3.

Second, the population-wide bacterial metabolic activity was assessed using a microcalorimetric assay that measures the heat flow. At increasing inoculum, the time to peak metabolic activity decreased significantly (*P* < 0.05; linear regression analysis [inoculum was log-transformed]). The time to peak of the lowest inoculum was 26.1 h ± 0.5 h, whereas peak metabolic activity was observed already after 11.0 h ± 1.0 h for the highest inoculum (Fig. 6A).

Last, to investigate if the metabolic activity of the different inoculums corresponded with a change in RNA content, we extracted RNA from alginate beads with PAO1 incubated for 24 h. The RNA content was correlated with paired CFU measurements, and for inoculum 1.0 and 0.01, we measured a very similar RNA concentration per CFU, whereas a 2-fold increase in RNA content per CFU was observed for inoculum 0.0001 (Fig. 6B).

### Differences in metabolic activity affect antibiotic susceptibility experiments.

To test whether the difference in metabolic activity after 24 h of incubation affected tolerance toward antibiotics, we exposed alginate beads with the highest and lowest inoculum to 10 and 50 μg mL^−1^ tobramycin, corresponding to 10× and 50× the MIC (Fig. 6C). The number of viable bacteria in the untreated controls was not significantly different, meaning that similar amounts of bacteria were exposed to tobramycin regardless of the inoculum. Fewer bacteria survived in the low inoculum than in the highest inoculum (*P* < 0.05) at both 10 and 50 μg mL^−1^ tobramycin. The effect was most pronounced when treated with 50 μg mL^−1^ tobramycin, where almost all bacteria in the low inoculum were killed, while 5.37 ± 0.39 log CFU mL^−1^ survived in the high inoculum.

## DISCUSSION

Microbiological experiments are often initiated based on tradition, and the choice of *in vitro* model, the starting concentration of bacteria, and which strains to select, often depend on routine standards, access to in-house models, or information from previous studies. However, initial decisions affect the outcomes in the selected model (24), and such an approach may lead to simplistic conclusions that are only valid under particular conditions. It has been previously demonstrated how different inoculation methods affect the size and frequency of aggregates in liquid batch cultures of P. aeruginosa and how this subsequently influences the tolerance toward tobramycin (21). In addition, recent work has revealed that the transcriptome of P. aeruginosa can change drastically between different *in vitro* models (24). Here, we report how the initial concentration of bacteria shapes the size and distribution of aggregates in the alginate bead biofilm model and how this influences phenotypic traits.

We show that modulation of the inoculum concentration alters the biomass distribution and the size of developed aggregates after a 24-h incubation. However, after 24 h of incubation, the total biomass was not different, even upon a 10^4^-fold increase in the inoculum. Enumeration of CFU was performed at 0 h, 24 h, and 48 h after inoculation, and thus that the precise development of biomass is missing. However, the microcalorimetric assay showed a time shift in peak metabolism between the different inoculums. Furthermore, the O_2_ consumption rates suggest the same pattern where the subsurface O_2_ consumption of the highest inoculums decreased toward zero after 8 h. Bacteria in the lowest inoculums sustained subsurface O_2_ consumption rates throughout the 24-h experimental period, leading to larger aggregates in the models’ interior. Thus, the bacteria in the highest inoculums are mainly constrained to the model edge with high competition for space and substrate, and the higher inoculums progress into the stationary growth phase earlier than lower inoculums (Fig. 5 and 6). However, we suspect that over time the O_2_ consumption profile of the low and high inoculums would be similar given that the concentrations of bacteria were similar after 24 h and 48 h, although the biomass distribution was radically different. Whether the large aggregates observed in the low inoculum beads are large enough to create self-imposed physiological gradients on an aggregate scale was not investigated here, but would be an avenue for future experiments.

In this study, the metabolic activity of the bacteria was reflected by the O_2_ consumption, as no other respiratory electron acceptors were supplied. Facultative anaerobes can grow on other electron acceptors (such as nitrate), albeit with a lower biomass yield on substrate and associated lower growth rates (7, 25–27). Provision of such alternative electron acceptors has previously been shown to modulate the biomass distribution in alginate beads (23).

The low susceptibility of biofilms toward antibiotics is multifactorial (28, 29). It can be acquired by mutations or horizontal gene transfer (then termed resistance) or be determined by environmental factors such as O_2_ limitation (16) and diffusion retardation (30), and other matrix effects (31). Finally, phenotypic factors such as transcriptional activation of efflux pumps (32) and low metabolic activity can lead to increased tolerance (33, 34). Recently, it was shown that otherwise susceptible Escherichia coli became highly tolerant to Cecropin A in the early stationary phase (35), indicating a rapid, growth-rate-dependent shift in antibiotic susceptibility. Previously it was demonstrated that the transition from the log to the stationary phase is associated with highly decreased oxygen consumption (36). Thus, the O_2_ concentration and microcalorimetric activity measurements indicate that a time shift in the arrival at the stationary phase occurred as a function of the inoculum (Fig. 4–6). While the highest inoculums displayed decreased subsurface O_2_ consumption, the lowest inoculums continually increased their consumption (Fig. 5F). This could indicate that they did not reach the stationary phase within 24 h of incubation, and peak metabolic activity was indeed observed after 26 h (Fig. 6A). Thus, as expected we measured high RNA content in the lowest inoculum after 24 h of incubation, indicating increased metabolic activity compared with the other inoculums (Fig. 6B). We do note, however, that microcalorimetry was performed in closed, static vials whereas O_2_ measurements and RNA quantification was performed on beads that had been incubated in an open system under shaking. Thus, the time points are not directly comparable, although the same patterns were observed.

Here, we challenged the highest and lowest inoculum of the alginate beads with tobramycin, as these inoculums showed the most significant differences in metabolic activity, albeit having the same number of viable bacteria. When treated with 10 and 50 μg mL^−1^ tobramycin, the lowest inoculum showed a higher reduction of surviving bacteria than the highest inoculum. This demonstrates that the metabolic activity of the bacteria determines the susceptibility toward tobramycin, as has been previously shown (15). Similarly, previous studies have shown that breaking up aggregates and re-inoculating them in fresh media increased the efficacy of tobramycin, which targets metabolic active bacteria, but not colistin, which targets slow-growing bacteria (37).

Thus, the outcome of an antibiotic susceptibility test using the model described here could yield different inhibitory concentrations of antibiotics, further emphasizing a careful selection of the initial inoculum concentration. The effect of arrival at stationary phase on antibiotic susceptibility has previously been demonstrated in planktonic cultures (38); however, here we show the same feature on developing biofilm aggregates.

Apart from the mechanisms of increased antibiotic tolerance, other essential aspects can be influenced by the dynamics in biomass distribution shown here. For example, the bacterial transcriptome would differ in oxic versus hypoxic/anoxic conditions. Bacterial transcriptomes from wounds with low O_2_ availability revealed an Anr-mediated hypoxia stress response and expression of genes associated with stationary-phase growth (39).

The dependence of density on bacterial physiology has been studied intensively, where, e.g., bacterial communication using a quorum sensing (QS) system is tightly linked to the density of bacteria. In a synthetic cystic fibrosis sputum media (SCFM2), it was thus shown that only aggregates containing ≥5,000 P. aeruginosa could signal neighboring aggregates, while smaller aggregates did not engage in interaggregate signaling (40). They used a sophisticated 3D-printing platform in combination with the complex SCFM2 media (41). We propose that using the model described here, similar experiments can be performed where bacterial aggregate sizes can easily be manipulated by changing the inoculum concentration and/or electron acceptor availability during aggregate formation (23).

Emerging evidence suggests that bacterial behavior is controlled by their microenvironment and that this can be very heterogeneous in biofilms and infections (12, 16, 42). Traditional microbiological research has mainly used shaken cultures, where bacteria are kept in suspension, and all bacteria experience similar conditions, although it has been shown that even vigorously shaken cultures contain aggregates (21, 37, 43). It has been shown that biofilms act as individual pharmacological compartments (44, 45), and a conceptual zone model was recently proposed for understanding bacterial behavior in relation to their microenvironment in infections (46). This scale-based model differentiates between the zones of single bacteria, the biofilm, the environment immediately around the aggregate, and the surrounding inflamed tissue. The analogy of this conceptual model to the alginate beads model is striking, and it is intriguing whether these models can successfully be modulated to better emulate bacterial behavior and host-bacteria interactions observed in infections. The rapid emergence of *in vivo* transcriptomic, proteomic, and metabolomic data (47–50) from infected patients will be able to guide us in the right direction of which factors are essential in such infections and the microenvironmental characteristics associated with them.

In conclusion, we show that the inoculum can lead to different phenotypical outcomes following a standard 24-h incubation period. Our results provide a framework for manipulating aggregate sizes in *in vitro* biofilm models. Further, they underline the importance of considering experimental procedures starting from bacterial inoculation, and we show that minor differences can lead to different microenvironmental characteristics and demonstrate their direct implications on the outcome of an antibiotic susceptibility experiment.

## MATERIALS AND METHODS

### Bacterial strain and media.

The bacterial strain P. aeruginosa PAO1, obtained from the Pseudomonas Genetic Stock Center (strain PAO0001) and tagged with a green fluorescent protein (GFP) expressed on plasmid pMRP9 (51), was used in this study. PAO1 was propagated from a −80°C freeze culture and grown overnight (ON) on Lysogeny broth (LB) agar plates. Single colonies were picked with an inoculation loop and grown ON in cell culture tubes with 5 mL media under continuous shaking (180 rpm; 37°C). Alginate beads bacteria were grown in low nutritional R2A broth supplemented with 0.05 M Tris-HCl buffer (adjusted to pH 7.6 with HCl) and 0.5% glucose, henceforth mentioned as R2A.

### Alginate bead preparation.

Alginate beads containing bacteria were prepared as previously described (23). PAO1 was inoculated in alginate beads using seaweed alginate (Protanal LF 10/60 FT; FMC Biopolymer, Drammen, Norway). Then, 9.5 mL 2% alginate was mixed with 0.5 mL ON culture OD_450_ adjusted to reach final optical densities in the beads of 0.1, 0.01, 0.001, and 0.0001. For the starting inoculum of OD_450_ = 1, a 4% alginate solution was mixed 1:1 with bacterial culture adjusted to OD_450_ = 2 to reach the same alginate concentration. OD_450_ from each cell culture tube was measured in triplicates, and the average was used. The alginate-bacterial solution was transferred to a 20 mL syringe connected to a 21 G hypodermic needle through a sterile silicone tube (internal diameter = 2 mm). The needle was placed 4.5 cm above the surface of a stirred 0.25 M CaCl_2_ solution and 12 mm from the edge of the beaker. Droplets of the alginate-bacteria solution were dispensed at 30 mL h^−1^ via a syringe pump (Perfusor Compact, Braun, Germany) into the CaCl_2_ solution and hardened for 1 h, and then washed three times in 0.9% NaCl. Twenty beads were gently transferred with sterile plastic tweezers to a 250-mL Erlenmeyer flask containing 50 mL R2A and incubated at 37°C, 100 rpm.

### Viable cell counts.

For viable cell counts of alginate beads, two beads were dissolved by whirl mixing in a 0.05 M Na2CO3/0.02 M citric acid solution for 10 min at 1,400 rpm. The alginate-bacterial slurry was subsequently sonicated in an ultrasonic water bath (5 min degas + 5 min sonication; Bransonic Ultrasonic Cleaner 2510, Emerson Electric, USA) before being serially diluted in 0,9% NaCl for enumeration of CFU per mL. Each dilution was spotted in triplicates (spot volume 10 μL) on LB plates following incubation for 24 h at 37°C. Samples were taken after 0 h, 24 h, and 48 h of incubation.

### Microscopy and image analysis.

Aggregates were visualized with a confocal laser scanning microscope (CLSM) (Zeiss LSM880). Alginate beads were cut in half with a scalpel and transferred to a microscope slide, and images were taken as tile scans from the edge toward the center of the bead with a plan-apochromat 20×/0.8 M27 objective. Each tile was recorded as z-stacks in 1.8-μm increments.

Quantitative image analysis was made in Fiji (ImageJ, version 2.1.0/1.53c). For the quantification of aggregate volumes, we used the “BoneJ” particle analyzer plugin (52). Brightness and threshold were globally adjusted, and a size threshold of 10 μm^3^ was chosen to exclude noise. Images were cropped to an x, y, and z size of 1,500 × 425 × 50 μm.

### O_2_ measurements.

Vertical profiles of O_2_ concentration through the alginate beads were measured using Clark-type O_2_ microelectrodes (tip diameter = 25 μm, OX-25, Unisense A/S, Aarhus, Denmark) with fast response time (<0.5 s) and low stirring sensitivity (<1% to 2%) (53), connected to a pA-meter (Unisense A/S, Aarhus, Denmark) and interfaced to data acquisition software (Profiling, Unisense, Aarhus, Denmark). Sensor signals were linearly calibrated at experimental temperature and salinity from measurements in aerated water and in water deoxygenated by the addition of sodium dithionite (Na_2_S_2_O_4_). Depth profiles of O_2_ concentration were measured in 0.1-mm increments from above the models toward the bottom. O_2_ profiles were measured in triplicates at 0 h, 4 h, 8 h, and 24 h after inoculation.

### O_2_ calculations.

Net consumption rates of O_2_ were calculated from fluxes of the measured O_2_ profiles using a modified version of Fick's first law of diffusion (54, 55).

$$J=0.5\left(-D\frac{C_{a}-C_{b}}{X_{a}-X_{b}}\right)+0.5\left(-D\frac{C_{b}-C_{c}}{X_{b}-X_{c}}\right)$$

where J is the flux of O_2_ (in nmol O_2_ cm^−2 ^min^−1^) through points a, b, and c, and D is the diffusion coefficient of O_2_ (2.5·10^−5^ cm^2^ s^−1^), C is the concentration of O_2_ (in μM), and X is the depth relative to the surface of the models (in μm). Calculations of surface and subsurface O_2_ consumption rates were done from O_2_ concentrations measured at depths of −200 to 0 μm and 100 to 300 μm, respectively.

### Microcalorimetric measurement of heat flow.

Measurements of heat flow (in μW) were obtained using a calScreener (Symcel, Sweden). Alginate beads were constructed as described above and adjusted to a final OD_450_ of 1, 0.1, 0.01, 0.001, and 0.0001. Single beads were positioned in sterile plastic inserts, filled with 200 μL R2A medium, and positioned in the sealed titanium cups. A 30-min preheating period was applied according to manufacturers’ guidelines. After preheating, samples were loaded into the measurement position, and signals were recorded at 1 Hz.

Raw thermograms were baseline corrected by subtracting the stable signal after all metabolic activity had ceased, and the initial thermal equilibration period was subtracted according to the manufacturer’s guidelines. Thermograms were analyzed by noting the time at which metabolic activity was highest (time to peak).

### Antibiotic treatment of alginate beads.

As described above, alginate beads were constructed and adjusted to a final OD_450nm_ of 1 and 0.0001. Before treatment, beads were incubated for 24 h in 100-mL Erlenmeyer flasks containing 10 mL R2A media. After 24 h of incubation, beads were treated with 0, 10, or 50 μg mL^−1^ tobramycin (Sigma) for 24 h. After exposure, two beads from each treatment were dissolved in a 200-μL solution of 0.05 M Na_2_CO_3_/0.02 M citric acid for 10 min. at 1,400 rpm. To wash out the tobramycin, 800 μL 0.9% saline was added to the alginate/bacteria slurry and centrifuged at 1,000 g for 5 min. The supernatant was removed, and the pellet was resuspended in 200 μL 0.9% saline before serial dilution and plating to obtain CFU counts.

### RNA extraction.

Alginate beads with PAO1 were constructed as described above and adjusted to a final OD_450nm_ of 1, 0.01, and 0.0001. Twenty beads were incubated as described above, and after 24 h of incubation, 10 beads were isolated for RNA extraction from each inoculum. The isolated beads were placed in a MagNA Lyzer (Roche) and homogenized for 3 × 30 s in 1 mL TRIzol Reagent (Invitrogen) with 1% β-mercaptoethanol. The samples were cooled down on ice for 1 min between each homogenization step. Next, 200 μL chloroform per 1 mL TRIzol Reagent was added, and the tubes were shaken for 45 s and incubated on ice for 5 min, followed by centrifugation for 30 min at 8,000 g. The upper aqueous layer was transferred, and 0.5 mL isopropanol per 1 mL TRIzol Reagent was added together with 2 μL linear acrylamide. Tubes were precipitated for 2 h at −20°C, followed by centrifugation for 30 min at 8,000 g at 4°C. The pellet was washed 2 times with 80% ice-cold ethanol, air-dried for 10 min, and resuspended in 50 μL of RNases-free water.

DNase treatment was performed with RNA Clean & Concentrato-5 kit (Zymo Research) according to the manufacturer’s description. Total RNA yield and possible DNA contamination were evaluated using a NanoDrop spectrophotometer (Thermo Fisher Scientific) and a Qubit fluorometer (Thermo Fisher Scientific).

### Plotting and Statistics.

Statistical tests and graphs were made with Prism 8.4 (GraphPad Software, La Jolla, USA) or R Statistical Software (version 3.6.3; R Foundation for Statistical Computing, Vienna, Austria). Isopleths of O_2_ concentration as a function of depth and time were plotted in R using the Plotly package. A *P* value <0.05 was considered statistically significant. CFU/mL was log-transformed before being plotted and was analyzed using a two-way ANOVA with Dunnet multiple comparison corrections. All experiments were carried out in at least biological triplicates.

### Data availability.

The data generated in this study are available from the corresponding author on reasonable request.
